# Biofilm formation, hemolysin production and antimicrobial susceptibilities of *Streptococcus agalactiae* isolated from the mastitis milk of dairy cows in Shahrekord district, Iran

**Published:** 2013

**Authors:** Azizollah Ebrahimi, Azar Moatamedi, Sharareh Lotfalian, Pejhman Mirshokraei

**Affiliations:** 1 *Department of Pathobiology, School of Veterinary Science, Shahrekord University, Shahrekord, Iran;*; 2 *DVM student School of Veterinary Science, Shahrekord University;*; 3 *Department of Clinical Science, School of Veterinary Science, Shahrekord University, Shahrekord, Iran.*

**Keywords:** Antimicrobial susceptibility, Biofilm, Iran, Mastitis, *Streptococcus*

## Abstract

*Streptococcus agalactiae* is a major contagious pathogen causing bovine sub-clinical mastitis. The present investigation was carried out to determine some phenotypic characteristics of the *S. agalactiae* strains isolated from bovine mastitis cases in dairy cows of Shahrekord in the west-center of Iran. One hundred eighty California mastitis test (CMT) positive milk samples were bacteriologically studied. A total of 31 (17.2%) *S. agalactiae* isolated. Twenty eight (90.3%) of the isolates were biofilm producers. This finding may indicate the high potential of pathogenicity in isolated strains. Sixteen (51.6%) isolates were α hemolysin producers. Only 19.3%, 22.5% and 29.0% of the isolates were sensitive to streptomycin, flumequine and kanamycin, respectively. None of these three agents is recommended for treatment of mastitis cases.

## Introduction

*Streptococcus agalactiae* is a highly contagious, obligate bacterium of the bovine mammary gland. This bacterium can survive a very short time in the environment, but it can persist indefinitely within the mammary gland as an obligate pathogen of the udder.^1^

The routine method for detection of *S. agalactiae* in milk samples is bacteriological culture. *S. agalactiae *is identified in the veterinary laboratory by Christie, Atkins, Munch, Petersentest (CAMP) factor, hydrolysis of hippurate and lack of hydrolysis of esculin. Carbohydrates fermentation is also used for confirming differentiation of *S. agalactiae *from other streptococci isolated from bovine mammary glands.^2^

The type of hemolysis produced by streptococcal species can be variable; β hemolysis defined as a clear zone of hemolysis around the colony, α hemolysis causes zone of greening or partial hemolysis and γ causes no visible hemolysis. Generally the β - hemolytic streptococci tend to be the most pathogenic for animals.^2^

The biofilms is consisted of micro colonies encased in extracellular polysaccharide material. Bacteria inside biofilms have increased resistance to antimicrobial agents. The production of biofilms was correlated with pathogenicity and virulence of bacteria.^3^

Many common bacterial pathogens exist in animals as biofilms. Mastitis is a typical animal disease where bacterial biofilms are believed to be involved based on histopathologic and ultrastructural appearance of the bacteria within tissue.^4^

To treat clinical mastitis successfully and to implement dry cow therapy efficiently, one should know the prevalence and the antimicrobial susceptibility of microorganisms isolated from the mammary gland. The susceptibility of bacteria in various geographical locations may change with time due to selective pressure of antimicrobial therapy. 

The present study was undertaken in order to determine some phenotypic characteristics of *S. agalactiae* isolates from bovine mastitis in Shahrekord district in the west centre of Iran. 

## Materials and Methods

**Sample collection. **The study covered a 10-month period from March to December 2011. Mastitis affected cows were identified by CMT (2+ or more).^5^ After pre-milk hand-stripping, teat orifices were scrubbed with a cotton pledged saturated in 70% ethyl alcohol.

CMT positive milk samples were aseptically collected from different dairy farms in Shahrekord district. The ice box containing milk samples in tubes were sent to the microbiology laboratory of veterinary school (Shahrekord University, Shahrekord, Iran) for isolation of strains. In total 180 CMT positive milk samples were collected.

**Isolation of** ***S. agalactiae. ***On arrival at the laboratory, 0.1 mL of milk were streaked onto thallium sulphate-crystal violet-B toxin blood agar (TKT agar; Merck, Darmstadt, Germany) medium containing 7% sheep blood, and incubated aerobically at 37 ˚C for 24-72 hr. Colonies with morphology matched to those of streptococci colonies,^2^ on each cultured plate were selected for further examinations. Colonies yielding gram-positive cocci with catalase-negative and oxidase negative reaction were pure cultured on sheep blood agar plates (Merck, Darmstadt, Germany), Hemolysis was scored and then subjected to CAMP test on sheep blood agar and esculin hydrolysis on TKT tests. Rapid hippurate hydrolysis test was conducted as described by Marcia and Grace.^6^ The growth of isolates on MacConkey agar (Merck, Darmstadt, Germany) were also examined. 

Carbohydrate utilization was conducted in phenol red broth (PRB; BBL Microbiology Systems, Cockeysville, USA) with 1% final concentration of the following carbohydrates: lactose, maltose, mannitol, raffinose, salicin and trehalose. Positive reactions were indicated by a change from red to yellow after aerobic incubation at 37 ˚C for 24 hr. Identification of *S. agalactiae* was performed as described by Quinn *et al*.^2^

**Biofilm assay. **The biofilm assay was performed by using micro titer plates as described by Tendolkar *et al*.^7^ Flat-bottom micro titer plates were used. *S. agalactiae* isolates were grown at 37 ˚C in tryptic soy broth (TSB; Merck, Darmstadt, Germany). The bacterial cells were then pelleted at 6,000 *g* for 10 min, and the cell pellet re-suspended in 5 mL of fresh medium. The optical densities (ODs) of the bacterial suspensions were measured using spectrophotometer (Model 6305, Jenway Ltd., Essex, UK) and normalized to an absorbance of 1.00 at 595 nm. The cultures were diluted 1:40 in fresh TSB and 200 μL of cells were dispensed into 12 wells in a single row of a sterile 96-well flat-bottom polystyrene micro titer plate. 

After incubation at 37 ˚C for 24 hr, the planktonic cells were aspirated and the wells washed three times with sterile phosphate-buffered saline (PBS). The plates were inverted and allowed to dry for 1 hr at room temperature. For biofilms quantification, 200 μL of 0.2% aqueous crystal violet solution was added to each well, and the plates were allowed to stand for 15 min. The wells were subsequently washed three times with sterile PBS to wash off the excess crystal violet. Crystal violet bound to the biofilms was extracted with 200 μL of an 80:20 (v/v) mixture of ethyl alcohol and acetone, and the absorbance of the extracted crystal violet was measured at 595 nm.

As a control, crystal violet binding to wells was measured for wells exposed only to the medium with no bacteria. All biofilm assays were performed in triplicate, with 12 replicates for each strain per assay.

Interpretation of biofilm production was according to the criteria described by Stepanovic *et al*.^8^ Based on these criteria optical density cut-off value (ODc) was defined as:

average OD of negative control + 3 × SD (standard deviation) of negative control, and the biofilms producers are categorized as: no biofilm producer ≤ ODc, weak biofilm producer ODc < ~ ≤ 2 × ODc, moderate biofilm producer 2 × ODc < ~ ≤ 4 × ODc and strong biofilm producer > 4 × ODc; where "~ " stands for average of sample ODs.

**Susceptibility testing. **For susceptibility testing, isolates were incubated in trypticase soy broth at 37 ˚C for 24 hr and the suspension was adjusted to a turbidity equivalent to a 0.5 McFarland standard. Susceptibility to antimicrobial agents was determined for isolated strains by the disk diffusion method on Mueller-Hinton agar (Merck, Darmstadt, Germany), containing 3-5% ovine serum, according to the National Committee for Clinical Laboratory Standards guidelines.^9^ The selected antibiotics for antibiogram were those that were more common in treatment of regional bovine mastitis cases as appeared in Table 1.

Isolates were categorized as susceptible and resistant based upon interpretive criteria developed by the National Committee of Clinical Laboratory Standards.^10^

**Table 1:** Antibiotic susceptibility responses of S. *agalactiae* strains isolated from CMT positive bovine milk samples.

**Antibiotics**		**Susceptibility**		
		**Sensitive** **(%)**	**Intermediate** **sensitive (%)**	**Non-sensitive** ** (%)**
**Amoxicillin** **Kanamycin** **Ampicillin** **Enrofloxacin** **Penicillin** **Ciprofloxacin** **Trimetoprime** **Tetracycline** **Gentamicin** **Flumequine** **Erythromycin** **Streptomycin**	30 (96.7) 09 (29.0) 30 (96.7) 19 (61.2) 23 (74.1) 28 (90.3) 25 (80.6) 27 (87.0) 23 (74.1) 07 (22.5) 19 (61.2) 06 (19.3)		00.0 (0)000) 05 (16.1) 00.0 (0)000) 12 (38.7) 2 (6.4) 3 (9.6) 1 (3.2) 1 (3.2) 3 (9.6) .0 (0)0) 2 (6.4) 07 (22.5)	1 (3.2) 17 (54.8) 1 (3.2) .0 (0)0) 06 (19.3) 0 (0)… ...5 (16.1) 3 (9.6) 05 (16.1) 24 (77.4) 10 (32.2) 18 (58.0)

## Results

Out of the 180 CMT positive milk samples studied for *S. agalactiae* infection, the bacteria was isolated from 31 (17.2%) of the milk samples. Twenty eight (90.3%) of the isolates were biofilm producers, among them 6 (19.3%), 14 (45.1%) and 8 (25.8%) isolates were strong, moderate and weak biofilm producers, respectively.

Sixteen (51.6%) isolates were α-hemolysin producers while we did not detect any β hemolysin one. Fifteen isolates (93.7%) of α hemolysin producer were biofilm producers simultaneously.

The sensitivity of the *S. agalactiae* isolates to anti-microbial compounds is given in Table 1. Overall, only 19.3%, 22.5% and 29.0% of the isolates were sensitive to streptomycin, flumequine and kanamycin, respectively.

## Discussion

In this study 31 (17.2%) isolates from 180 CMT positive milk samples were identified as *S. agalactiae.* This finding indicates that the prevalence of *S. agalactiae* in infected cows in Shahrekord district is almost the same as other regions of our country.^11^

Sixteen (51.6%) of our isolates were α hemolysin producers, whose 15 (93.7%) were also biofilm producers. Nicky and O’Toole showed a role for α hemolysin in *S. aureus* biofilm formation and that this toxin appears to be required for cell-to-cell interactions.^12^ This role may also be involved in *S. agalactiae* but we could not find similar reports in this regard.

We did not detect any β hemolysin producer isolates. In bovine strains of *S. agalactiae* the type of hemolysis produced can be variable but in human isolates it seems that production of β hemolysin is a constant feature.^2^^,^^13^ Twenty eight (90.3%) of our isolates were biofilm producers. The production of biofilms was correlated with pathogenicity and virulence of bacteria.^3^ Microorganisms inside biofilms have increased resistance to antimicrobial agents.^3^ Thus, our finding may indicate the high potential of pathogenicity in isolated strains. We could not find reports regarding the prevalence of biofilm formation among the strains of *S. agalactiae*, but it is documented that it has the potential of producing this virulence factor.^4^ Comparing to other streptococci, Petersson-Wolfe *et al*. reported a low frequency of biofilm formation by *E. fecalis *isolates*.*^14^ They concluded that biofilm formation by this species did not seem to be a prerequisite for colonization of the bovine mammary gland. In other hand, biofilm formation by *S. pyogenes* is reported to be an important virulence factor.^15^

The ability of *S. agalactiae *to produce slime might be a desirable virulence factor during colonization of the udder. It has been shown that slime production is important, allowing some bacteria to aggregate and form biofilms.^16^

The antimicrobial susceptibility data for isolates of *S. agalactiae* are summarized in Table 1. Results of this study demonstrated low sensitivity for streptomycin, flumequine and kanamycin. These agents are used to treat mastitis cases and other diseases in cattle. None of these three agents is recommended for treatment of mastitis cases due to their limited activity against the examined organisms. 

Some authors documented resistance to streptomycin, kanamycin and sensitivity to β-lactam drugs in *Streptococcus *species isolated from clinical mastitis in dairy cows,^17^ our results are in line with these findings. 

In summary, the majority of* S. agalactiae isolates* (90.3%) evaluated in this study formed biofilms under *in vitro* conditions. Further work to assess *S. agalactiae* ability to form biofilms *in vivo* and intra mammary infection is suggested. This will allow for development of new strategies to a better management and prevention of mastitis caused by this pathogen. The isolates also showed low sensitivity to streptomycin, flumequine and kanamycin. More vigilant policies on the use of antibiotics in animals may limit distribution of resistance genes between bacteria and result in an improvement of the current situation. 
